# Investigation of the Temperature Fluctuation of Single-Phase Fluid Based Microchannel Heat Sink

**DOI:** 10.3390/s18051498

**Published:** 2018-05-10

**Authors:** Tao Wang, Jiejun Wang, Jian He, Chuangui Wu, Wenbo Luo, Yao Shuai, Wanli Zhang, Chengkuo Lee

**Affiliations:** 1 School of Electronic Science and Engineering, University of Electronic Science and Technology of China, 2006 Xiyuan Avenue, Chengdu 611731, China; wstudy92@163.com (J.W.); jackyhale@163.com (J.H.); cgwu@uestc.edu.cn (C.W.); luowb@uestc.edu.cn (W.L.); yshuai@uestc.edu.cn (Y.S.); wlzhang@uestc.edu.cn (W.Z.); 2 State Key Laboratory of Electronic Thin Film and Integrated Devices, University of Electronic Science and Technology of China, 2006 Xiyuan Avenue, Chengdu 611731, China; 3 Department of Electrical and Computer Engineering, National University of Singapore, 4 Engineering Drive 3, Singapore 117583, Singapore; elelc@nus.edu.sg

**Keywords:** single-phase microchannel heat sink, temperature fluctuation, thin film temperature sensors, microfluidics, temperature gradient

## Abstract

The temperature fluctuation in a single-phase microchannel heat sink (MCHS) is investigated using the integrated temperature sensors with deionized water as the coolant. Results show that the temperature fluctuation in single phase is not negligible. The causes of the temperature fluctuation are revealed based on both simulation and experiment. It is found that the inlet temperature fluctuation and the gas bubbles separated out from coolant are the main causes. The effect of the inlet temperature fluctuation is global, where the temperatures at different locations change simultaneously. Meanwhile, the gas bubble effect is localized where the temperature changes at different locations are not synchronized. In addition, the relation between temperature fluctuation and temperature gradient is established. The temperature fluctuation increases with the temperature gradient accordingly.

## 1. Introduction

With the development of microelectronics technology in the direction of miniaturization, integration, and intellectualization, the heat flux of microelectronic devices is increasing sharply [1,2]. Thermal management technology is becoming more important for the further development of microelectronic technology [2]. Based on the ever-growing demands for thermal management of electronic devices or chips, some promising liquid cooling techniques are proposed. Among them, the MCHS receives great attention from researchers around the world [3,4,5,6]. The latest research shows that the heat flux up to 910 W/cm^2^ is dissipated by a hierarchical manifold microchannel heat sink array [7]. Various structures of microchannel heat sinks are designed to find the optimization of its heat dissipation capacity, such as the single-layer (SL-MCHS) [8], double-layer (DL-MCHS), and multi-layer MCHS (ML-MCHS) [9,10], and great amounts of heat-transfer enhancement techniques are reported [11,12,13]. 

In particular, as a significant factor for the reliability and performance of the MCHS, temperature instability has drawn more attention in recent years [14,15]. Many researchers point out that the temperature stability of a microchannel is more susceptible than macro or conventional channels [16,17]. However, most of the studies focus on the boiling state, where bubble generation, growth, and departure contribute to temperature fluctuation [18,19,20]. The coolant in such studies is under subcooled or saturated flow boiling state, where the temperature is usually higher than 65 °C [16,21]. There are few studies about the temperature fluctuation of MCHS with single phase coolant.

In this paper, the MCHS temperature fluctuation under single-phase condition is studied. Deionized water is used as the coolant. Integrated thin-film temperature sensors developed by us are used for investigating the temperature fluctuation. Such sensors can provide direct and more accurate information inside the microfluidic channel [22]. A finite elements model (FEM) is also built to analyze the cause of temperature fluctuation. Results show that even when the coolant is under single phase, the temperature fluctuation in the microchannel is not negligible. The inlet temperature fluctuation and separate-out air bubbles both are the main causes, which contribute to the temperature fluctuation of MCHS. Furthermore, the relation between temperature fluctuation and temperature gradient is established. The temperature fluctuation is linearly dependent on the temperature gradient of the microchannel, where a larger temperature gradient leads to higher temperature fluctuation of the MCHS. The separate-out air bubbles, on the other hand, may further enhance the temperature fluctuation significantly.

## 2. Design Considerations and Measurement Setup

**Figure 1:**
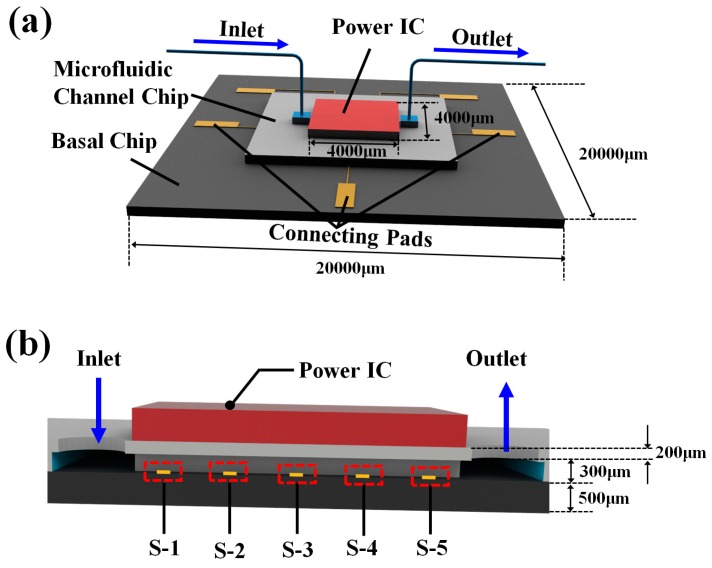
(**a**) Schematic drawings of microchannel heat sink structure and (**b**) cross-sectional view along the central microchannel, where the thin film temperature sensors are denoted as S-1 to S-5.

An MCHS with integrated thin-film temperature sensors is designed to investigate the temperature fluctuation, as shown in the Figure 1a.

The MCHS consists of three parts, including the power IC (working as heat source, and its dimensions are 4000 × 4000 μm), microfluidic channel chip, and basal chip. The microfluidic channel chip and basal chip are both made of silicon. The microfluidic channel chip consists of 50 rectangular micro channels, and the dimensions of each channel are 50 × 300 × 5000 μm (the width is 50 μm, the depth is 300 μm, and the length is 5000 μm). The micro channels are fabricated by dry etching. Five temperature sensors, denoted as S1 to S5, are equally distributed in the central fluidic channel from inlet to outlet. The interval of each sensor is 1000 μm, shown in Figure 1b. Pt thin-film is deposited and patterned as the temperature sensor. The benzocyclobutene (BCB) epoxy is used for chip bonding. After coating 2-μm BCB epoxy on the basal chip, a die-to-die bonding process is performed between the basal chip and microfluidic channel chip, and the thin-film temperature sensors are fully integrated into the MCHS. Compared with the sensors fabricated on the outside surface of MCHS, the temperature sensors integrated inner microchannel is more accurate and less affected by the environment [7,16,17]. Moreover, the BCB epoxy as the bonding layer also works as a protective layer, which prevents the Pt thin-film temperature sensors from contacting the coolant directly. Such a protective method ensures the reliability of the Pt temperature sensors. The excellent isolation of BCB epoxy also avoids the noise generated by the current leakage.

The temperature sensors are carefully calibrated by VPF-100-FTIR Cryostat after all the sensors are annealed at 350 ℃ for 1 hour. To precisely measure the resistance of the temperature sensor, all sensors are four-wire connected, eliminating the effect of parasitic resistance of the connection wires. Before calibration, the pressure in thermostatic chamber is maintained below 100 Pa (preventing the vapor in the air from freezing). After regulating the temperature in the cryostat by temperature controller, stabilized resistances of the sensors are recorded. The results of calibration show that the Pt temperature sensor maintains good thermal response and high linearity.

The power IC is driven by a DC power source, while an injection pump controls the coolant (deionized water) flow rate. All the sensors are connected to data acquisition system (DAQ), and a LabVIEW program is developed for automatic measurement and data process. The data acquisition interval is 6 seconds. The resistance changes are then transmitted to PC for temperature conversion, display, and storage. The transient temperature changes can be monitored in real time, and an infrared (IR) camera is installed over the MCHS and used to monitor the surface temperature of power IC.

The uncertainties of the measurements are shown as below:

(1) During the temperature calibration, the uncertainties related to temperature measurements are ±0.05 °C for the VPF-100-FTIR Cryostat. Therefore, the calibrated errors would influence the temperature fluctuation of the measurement.
(2) In the measurement, the data acquisition system (DAQ) connected to the temperature sensors is the Keithley 2700 data logging system. The resolution of the DAQ is 10^−6^ Ω. Based on the temperature coefficient of resistance (TCR), the sensors have an accuracy of ±1.04 × 10^−6^ °C using the present acquisition system.
(3) In order to minimize the effect of parasitic resistance of the connection wires, all the sensors are four-wire connected. Any temperature induced resistance change of the wires should have been eliminated. However, such elimination cannot be complete, and still some error could be introduced into the measurement results.

## 3. Results and Discussion

**Figure 2:**
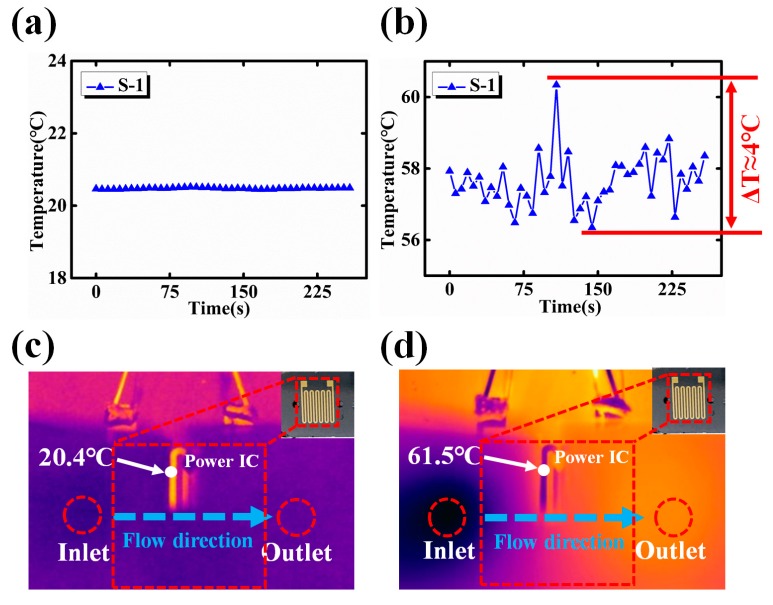
The temperature changes under the condition of (**a**) flow rate = 50 mL/h, input power = 0 W; (**b**) flow rate = 50 mL/h, input power = 4.2 W. Corresponding photographs of IR camera under the condition of (**c**) flow rate = 50 mL/h, input power = 0 W; (**d**) flow rate = 50 ml/h, input power = 4.2 W.

When the power IC is turned off and the coolant flow rate is fixed to 50 mL/h, the temperature change with respect to time is plotted in Figure 2a. This initial temperature fluctuation is very small (within 0.05 °C), which reveals the temperature of micro channel does not change much and the background noise of the testing setup is low. After the temperature in the microchannel is stable, an operating power (4.2 W) is applied to the power IC. As it is shown in Figure 2b, the temperature fluctuates within range of 4 °C The corresponding infrared pictures of power-off and power-on states are shown in Figure 2c,d, respectively. As shown in Figure 2d, the highest surface temperature of the heat source (resistor coil) is 61.5 °C, which is far from the boiling point of the coolant (deionized water). Therefore, even when the coolant is in single phase, the temperature fluctuation in the microchannel is not negligible.

**Figure 3:**
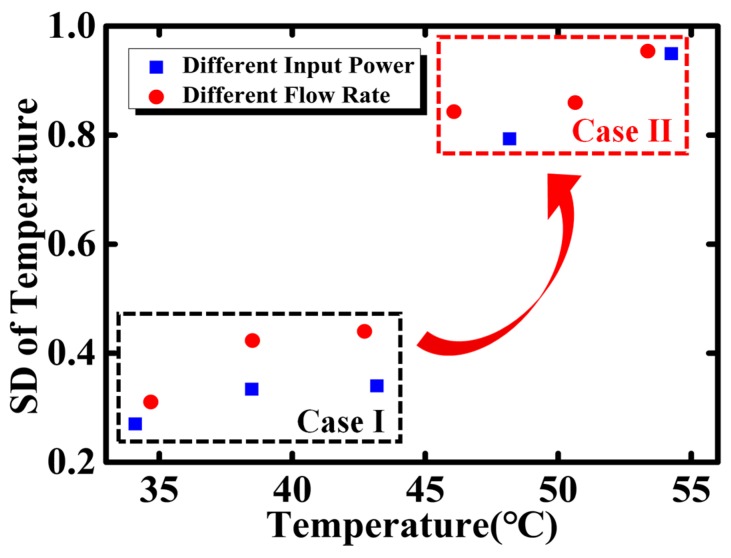
The calculated standard deviation of the temperature with the different input power (1.5, 2.0, 2.5, 3.0, and 4.0 W, but a constant flow rate 50 mL/h) and different flow rate (70, 80, 90, 120, 150, and 200 mL/h) but a constant heating power of 5.5 W.

In order to study the temperature fluctuation quantitatively, the standard deviation (SD) of the temperature is calculated by 80–100 data points, where the duration time is about 10 min. The SDs of temperature with constant input flow rate (50 mL/h) and constant input power (5.5 W) are extracted and plotted in Figure 3. The temperature fluctuation seems solely dependent on temperature, despite of the input power or coolant flow rate. In particular, an abrupt increment of SD occurs at 45 °C. This phenomenon reveals that the dominant mechanism contributing to the temperature fluctuation may be different. The SDs therefore are divided into two groups, denoted as Case I and Case II, where a large gap is observed between them.

**Figure 4:**
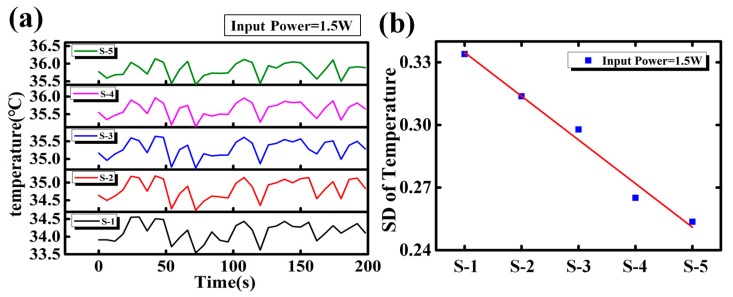
(**a**) Transient temperature responses at different locations for Case I (input power = 1.5 W) and (**b**) standard deviations of temperatures along the micro channel from inlet to outlet (S-1 to S-5).

In terms of Case I (below 45 °C), the transient responses of temperature at different locations of MCHS are shown in Figure 4a. Results show the temperatures at different locations are highly synchronized, where the temperatures at different locations vary simultaneously. Such synchronous phenomena reveal that the temperature fluctuation is not induced by localized cause, but a global cause, i.e., the cause could affect the whole MCHS. Figure 4b shows the SD decreases linearly from inlet to outlet (S-1 to S-5), implying the cause of temperature fluctuation may originate from the inlet. 

**Figure 5:**
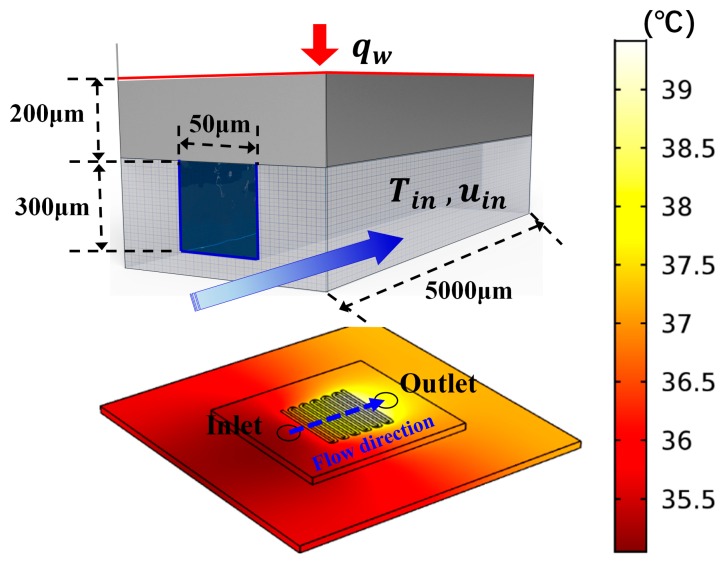
The simulation model of studying the temperature fluctuation in the MCHS.

In order to identify the main cause of temperature fluctuation, a finite element model (FEM) of the MCHS is built, shown in Figure 5. The schematic drawing of a unit microchannel is also shown in this figure. The model can be described using governing differential equations where the thermoelastic damping and viscous dissipation are neglected

(1) $$\rho C_{\rho }\frac{\partial T}{\partial t}+\rho C_{\rho }u\cdot \nabla T-k\nabla^{2}T=Q$$

where ρ is density, $C_{\rho }$ is heat capacity at constant pressure, u is flow rate of coolant fluid, T is temperature, k is conductivity, and Q is heat source. Considering the fluid flow, the dynamic momentum equation is expressed as

(2) $$\rho \frac{\partial u}{\partial t}+\rho \left(u\cdot \Delta \right)u=\nabla \left[-P+\mu \left(\nabla u+{\left(\nabla u\right)}^{T}\right)\right]$$

where P is pressure, and the continuity equation is

(3) ρ∇·u=0

Compared with the static modeling by Qu, W.L. et al. [23] and Li, J. et al. [24], the transient variables and operators $\frac{\partial T}{\partial t}$ and $\frac{\partial u}{\partial t}$, representing the transient change of temperature and fluid velocity, are taken into account to investigate the dynamic temperature fluctuation. $q_{w}$, $u_{in}$, and $T_{in}$ are the initial conditions, where $q_{w}$ is the power applied on the IC, $u_{in}$ the fluidic velocity from inlet, and $T_{in}$ the inlet temperature.

Simple variable method is employed for study. The sinusoidal factors are introduced to initial conditions to describe the fluctuation, and they are expressed as

(4) $$q_{w}=q_{w0}\cdot \left(1+10\%\cdot \sin\left(\omega_{0}t\right)\right)$$

(5) $$u_{in}=u_{in0}\cdot \left(1+10\%\cdot \sin\left(\omega_{0}t\right)\right)$$

(6) $$T_{in}=T_{in0}\cdot \left(1+10\%\cdot \sin\left(\omega_{0}t\right)\right)$$

**Figure 6:**
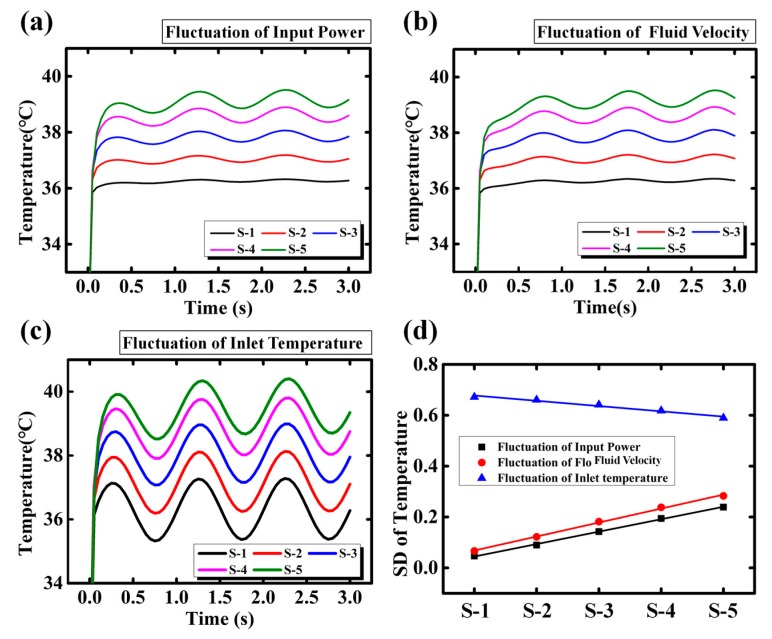
The simulation results of different sinusoidal single variances: (**a**) input power; (**b**) flow rate and (**c**) inlet temperature; (**d**) standard deviation of temperature fluctuation from inlet to outlet (S-1 to S-5) of different sinusoidal single variances.

The simulation results are plotted in Figure 6a–c respectively. Results show that the temperature fluctuations originated from $q_{w}$, $u_{in}$, and $T_{in}$ are all synchronized. The SDs of temperature are calculated and plotted in Figure 6d. It is clearly shown that only the temperature fluctuation originated from $T_{in}$ decreases linearly from inlet to outlet, which matches with the experimental results (Figure 4b). When the power IC (heater) is working, the heat generated from heater continually transfers to the inlet. The heat transfer coupled with the injected liquid at the inlet may be unstable. Such instability of heat results in the fluctuation of the inlet temperature. The temperature fluctuations originating from input power and fluidic velocity, however, accumulate along the microchannel. Actually, due to the thermal convection of the fluid, the heat accumulates downstream of the microchannel [23,24]. The accumulation of heat causes the downstream temperature rise [22]. When either the input power or the fluid velocity is fluctuating, the temperature in the microchannel fluctuates concomitantly and the downstream shows stronger fluctuation. This seems to contradict the experimental results. Therefore, the main cause of the temperature fluctuation for Case I should be attributed to the inlet temperature.

**Figure 7:**
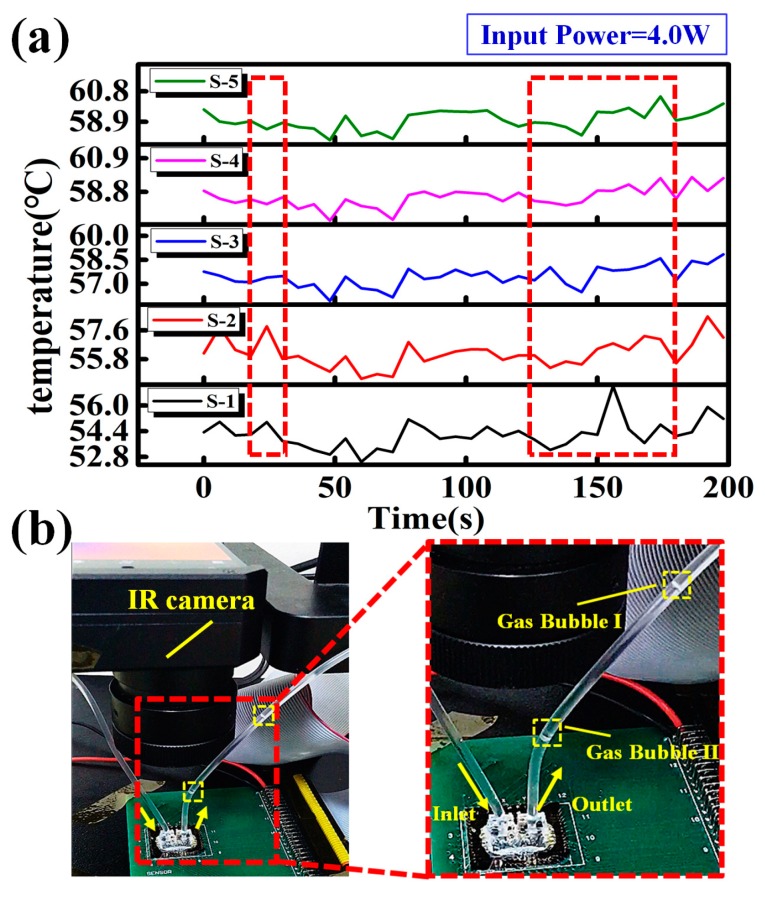
(**a**) Transient temperature responses at different locations for Case II (input power = 4.0 W) and (**b**) pictures of gas bubbles observed from the outlet.

For Case II (above 45 °C), the transient responses of temperature at different locations of MCHS are shown in Figure 7a. The temperature synchronization phenomenon is disrupted. In the meantime, gas bubbles are observed clearly from the outlet during the experiment, shown in Figure 7b. The random temperature fluctuation is likely to be caused by such gas bubbles. Since the gas bubbles keep discharging regularly from the outlet while no gas bubble enters the MCHS from inlet, these gas bubbles should be generated inside the MCHS. According to Henry’s Law [25], the maximum concentration of gas in the aqueous phase can be expressed as

(7) $$c_{a}=\frac{H^{cc}}{R}\cdot \frac{P_{g}}{T}$$

where $H^{cc}$, $P_{g}$, and R are the dimensionless Henry solubility, pressure of gas, and gas constant. Equation (7) reveals that the maximum amount of air dissolved in water increases with pressure $P_{g}$ and decreases with temperature T. When the pressure remains constant, the air solubility in water reduces as the temperature increases [25]. In this study, the MCHS is working in an open environment, and the pressure drop in the microchannel is measured about 15 kPa. Such a pressure drop is negligible compared to atmosphere pressure (101.325 kPa). Therefore, the temperature change is the dominant factor affecting the maximum air concentration in water. As the temperature increases, when the amount of air dissolved in water is higher than the corresponding $c_{a}$, the dissolved air in water separates out, forming large amounts of gas bubbles. Since the gas bubbles are not uniformly formed in the microchannel, the temperature at different locations may not vary simultaneously, i.e., the synchronization may be disrupted. 

**Figure 8:**
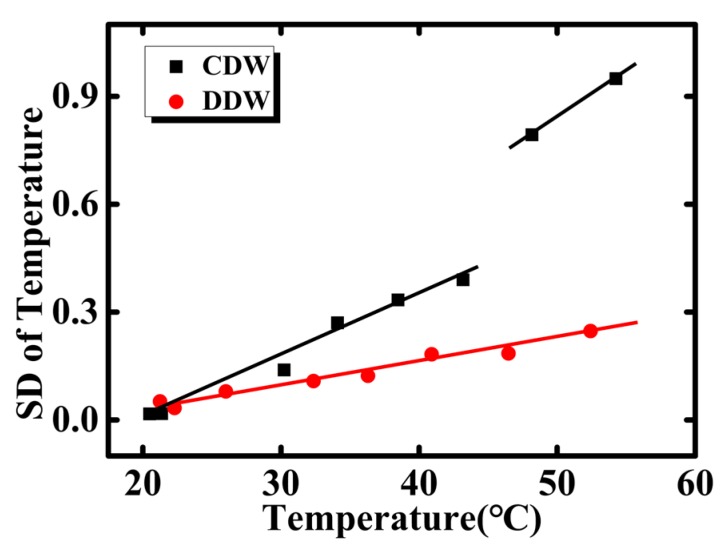
The standard deviation of temperature fluctuation change with temperature when using CDW or DDW as coolant.

A comparative experiment is conducted to further investigate the gas bubble effect, where the deaerated deionized water (DDW) is employed for a comparison to common deionized water (CDW). In order to deaerate the air dissolved in coolant, the DDW is fully boiled for 1 h and then vacuumed thoroughly. The coolant flow rate is set at a fixed value (50 mL/h). The SDs of temperature for CDW and DDW are calculated under different input power (the input power is 0, 0.1, 1.0, 1.5, 2.0, 2.5, 3.0, and 4.0 W), the results are plotted in Figure 8. When using the CDW as the coolant, the SD rises as the temperature, and abrupt change is observed at about 45 °C. On the contrary, the temperature for DDW is more stable. No sudden change of SD is observed even at 60 °C. 

In Henry’s law, the maximum concentration of gas in water ($c_{a}$) decreases monotonously with the increase of temperature ($c_{a}=\frac{H^{cc}}{R}\cdot \frac{P_{g}}{T}$). Here, $c_{a}$ is the saturated solubility of the gas in the aqueous phase. In this study, the real concentration of gas dissolved in deionized water does not reach the saturated solubility in a particular atmospheric pressure and temperature. When the temperature is below 45 °C, the real concentration of gas dissolved in deionized water is lower than the saturated solubility ($c_{a}$), and bubbles are nearly separated out. While the temperature increases over 45 °C, the real concentration of dissolved gas in deionized water is over the corresponding saturated solubility. Therefore, the dissolved gas in the water separates out and forms bubbles, which significantly increases the temperature fluctuations in the microchannel, and results in the abrupt change. For DDW, however, the gas is deaerated thoroughly, and the real concentration of gas dissolved in DDW is much lower than the $c_{a}$. Therefore, there are hardly any bubbles separated out, and no abrupt change is observed. Based on the above discussion, the separate-out gas bubbles from coolant (deionized water) is another factor that influences the temperature fluctuation in single phase. The bubble effect dominates at higher temperature.

**Figure 9:**
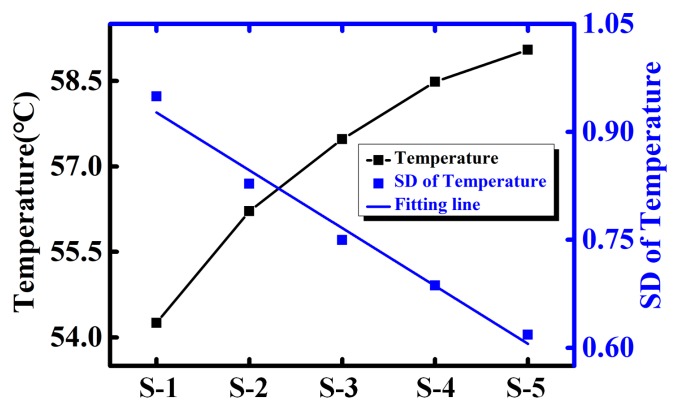
Temperature and temperature fluctuation change of CDW from inlet to outlet (S-1 to S-5) when flow rate is 50 mL/h and the input power is 4.0 W.

**Figure 10:**
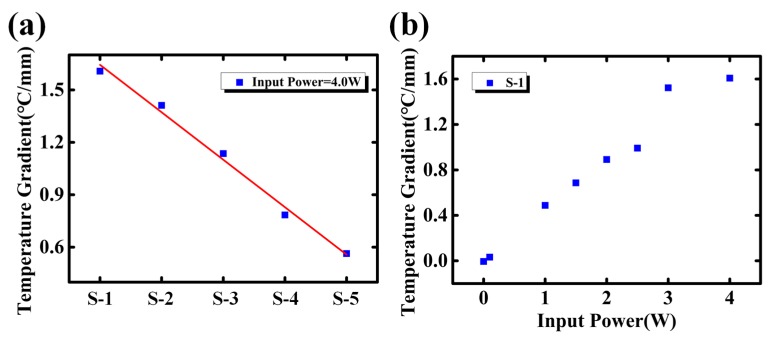
(**a**) Temperature gradient changes along the microchannel (S-1 to S-5); and (**b**) temperature gradient changes with different input power.

A noteworthy phenomenon is shown in Figure 9. As is discussed above, higher temperature should have brought in larger temperature fluctuation. However, the SD of temperature reduces while the temperature increases along the microchannel (from inlet to outlet). Hence, the temperature fluctuation may not be directly related to the temperature. As the temperature distribution along the microchannel is continuous, a continuous temperature curve is firstly obtained by the fitting function. As is shown in Figure 10a, the temperature gradient is calculated based on such continuous curve. Results show that the temperature gradient linearly drops from inlet to out, which matches well with the decreasing trend of SD. Moreover, as the input power increases, both the temperature and temperature gradient are lifted, shown in Figure 10b [22]. Therefore, the temperature fluctuation may be related to the temperature gradient rather than temperature itself. 

**Figure 11:**
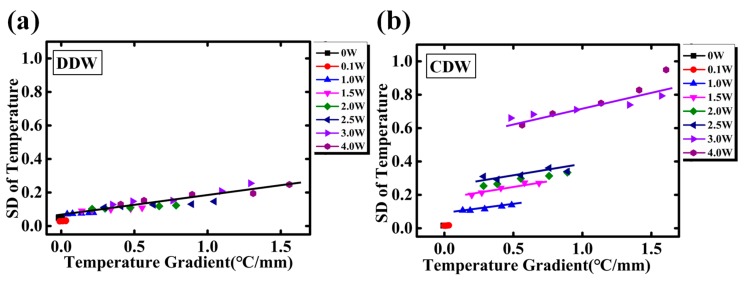
(**a**) DDW is used as coolant, the temperature fluctuation changes with temperature gradient at different input power and (**b**) CDW is used as coolant, the temperature fluctuation changes with temperature gradient at different input power.

The SDs of temperature (DDW as the coolant) with respect to temperature gradient is plotted in Figure 11a. Despite of the different input powers and different locations, all SDs obey the linear relation with temperature gradient. According to J. Lia et al. [24], the local heat flux is determined by the temperature gradient as q = −γ∇T(γ is thermal conductivity). Large heat flux is located where temperature gradient is high. Any temperature perturbation may break the heat equilibrium, and the following re-equilibrating process might bring in large temperature fluctuation in the microchannel. Further investigation of the mechanism of the temperature gradient related fluctuation will be our future work. Meanwhile, when using the CDW as coolant, the SDs with temperature gradient are described in Figure 11b. Results show the temperature fluctuation still increases linearly with the temperature gradient, and shares the same slope as that for DDW. However, the SD increases stepwise as the input power increases, and this is due to the gas bubbles separated out from CDW. Based on the above discussion, reducing the fluctuation of inlet temperature and the deaeration of coolant can reduce the temperature fluctuation in single-phase MCHS. Lowering the temperature gradient can also be an effective way to relieve the temperature fluctuation.

## 4. Conclusions

In this paper, the temperature fluctuation in single-phase microchannel heat sink have been investigated by the integrated temperature sensors. The conclusions obtained from this study are summarized as follows:

(1) Results show the temperature fluctuation in single phase MCHS is also not negligible. Moreover, the factors that resulted in the temperature fluctuation have been discussed and revealed based on simulation and experiment.
(2) According to the research, the fluctuation of inlet temperature and the separate-out gas bubbles dissolved in deionized water are both main factors affecting the temperature fluctuation.
(3) The fluctuation of inlet temperature is a global cause, meanwhile the gas bubbles separated out from deionized water change the temperature at random locations. The gas bubble effect becomes the dominant cause at higher temperatures.
(4) The temperature fluctuation is linearly dependent on the temperature gradient of the microchannel, where a larger temperature gradient leads to a higher temperature fluctuation of the MCHS.
